# Expression of Xenobiotic Biomarkers CYP1 Family in Preputial Tissue of Patients with Hypospadias and Phimosis and Its Association with DNA Methylation Level of SRD5A2 Minimal Promoter

**DOI:** 10.1007/s00244-017-0466-x

**Published:** 2017-10-27

**Authors:** Seiichiroh Ohsako, Toshiki Aiba, Mami Miyado, Maki Fukami, Tsutomu Ogata, Yutaro Hayashi, Kentaro Mizuno, Yoshiyuki Kojima

**Affiliations:** 1 Laboratory of Environmental Health Sciences, Center for Disease Biology and Integrative Medicine, Graduate School of Medicine, The University of Tokyo, Bunkyo-ku, Tokyo, 113-0033 Japan; 2 Department of Molecular Endocrinology and Metabolism, National Research Institute for Child Health and Development, Setagaya-ku, Tokyo, 157-8535 Japan; 3 Department of Pediatrics, Hamamatsu University School of Medicine, Hamamatsu, Shizuoka 431-3192 Japan; 4 Department of Nephro-Urology, Nagoya City University Graduate School of Medical Sciences, Nagoya, 467-8601 Japan; 5 Department of Urology, Fukushima Medical University School of Medicine, Fukushima, 960-1295 Japan

## Abstract

Several epidemiological studies have suggested that the incidence of male reproductive organ malformations, including hypospadias or cryptorchidism, has increased due to fetal-stage exposure to environmental pollutants. However, the association of chemical exposure with the expression of target regulatory genes in the tissues of patients has not yet been reported. Because experimental approaches or clinical trials in human studies are limited, especially those using fetal and/or infants, it is difficult to obtain clear physiological evidence of mechanisms underlying male reproductive malformations. Thus, the lack of physiological evidence makes this issue controversial. We analyzed preputial tissues from patients with hypospadias (*n* = 23) and phimosis (*n* = 16). The atypical CYP1 family genes, CYP1A1 and CYP1B1, are potential biomarkers of environmental chemical exposure. We then compared the expression levels of CYP1A1 and CYP1B1 between hypospadias and phimosis samples by quantitative RT-PCR analysis. The mRNA expression levels of SRD5A2 and AR also were measured, because the androgen-related genes involved in the onset of disorders of male reproductive system. A significantly higher CYP1B1 expression level and a lower AR expression level were observed in the hypospadias groups than in the phimosis group. Positive correlations (*P* < 0.001) between the mRNA expression levels of the CYP1 family and SRD5A2 were found in patients with hypospadias but not in those with phimosis. Moreover, the methylation levels of the four genes were determined by bisulfite genomic sequencing. Although the SRD5A2 promoter region showed moderate methylation, no methylation was detected in CYP1A1, CYP1B1, or AR. There was no significant difference in SRD5A2 promoter methylation level between hypospadias and phimosis patients. Negative correlations were found between the methylation level of SRD5A2, especially at the − 221 Sp1 site, and the CYP1 family mRNA expression levels (CYP1A1, *p* = 0.002; CYP1B1, *p* = 0.007) in hypospadias patients, but not in phimosis patients. The significant positive association of mRNA expression level and the negative association of methylation level of the SRD5A2 gene with the mRNA expression levels of CYP1 family genes in the preputial tissue seem to indicate the chemical exposure of patients with hypospadias.

**Electronic supplementary material:**

The online version of this article (doi:10.1007/s00244-017-0466-x) contains supplementary material, which is available to authorized users.

During the past century, the prevalence of male reproductive system abnormalities, especially hypospadias, has increased markedly in developed countries, such as the United States, Australia, and Europe (Paulozzi et al. 1997; Nassar et al. 2007; Toppari et al. 2010; Skakkebaek 2016; Skakkebaek et al. 2016). An epidemiological study indicated higher prevalences of hypospadias and cryptorchidism in the general population living in areas with extensive farming and pesticide use (Carbone et al. 2006). Thus, many researchers still suspect that exposure to environmental chemicals, such as endocrine disruptors, resulted in the increase in the prevalence of disorders of the male reproductive system (Kalfa et al. 2009; Skakkebaek et al. 2016). If environmental chemicals cause such malformations of male reproductive organs, exposure must occur during the fetal or neonatal period. However, the measurement of chemical contaminants is extremely difficult even when using blood samples because of limitations of sample volume and ethical hurdles in the case of infants.

Organochlorine compounds (OCs), polycyclic aromatic hydrocarbons (PAHs), and dioxin-like chemicals, such as 2,3,7,8-tetrachlorodibenzo-*p*-dioxin (TCDD), are typical ligands of the aryl hydrocarbon receptor (AHR), a ligand-dependent transcription factor, which can bind to the xenobiotic responsive element (XRE) located on the target gene promoter region (Denison et al. 1989; Esser and Rannug 2015). Liganded AHR directly associates with sex-steroid hormone receptors (SHRs), such as the estrogen receptor (ER) and androgen receptor (AR), in the nucleus. By occupying the SHR-binding domain of DNA, a liganded AHR-SHR complex activates transcription, thereby disrupting sex-hormone-dependent transcription (Ohtake et al. 2003). Because cytochrome P450 1 (CYP1) family genes, such as CYP1A1 and CYP1B1, are the classical targets of AHR, the level of exogenous chemical exposure reflects the expression levels of CYP1 family genes in many tissues of rodents (Lubet et al. 1992) and probably in human epidermal cells (Katiyar et al. 2000; Yengi et al. 2003). Because it is very difficult to measure xenobiotic compounds using minute amounts of clinical samples, the mRNA expression levels of CYP1 family members by sensitive RT-qPCR will be a good alternative because the expression levels correlate with the level of endocrine disruption.

5α-Reductase type 2 (SRD5A2) deficiency causes the 46, XY disorder of sex differentiation (Imperato-McGinley et al. 1974). SRD5A2 polymorphism has been well documented to be positively associated with the onset of hypospadias (Thai et al. 2005; Maimoun et al. 2011), as well as the other causative gene MAMLD1 (Fukami et al. 2006; Ogata et al. 2012). In rodent experiments, low-dose TCDD exposure causes developmental abnormalities in nascent male genital organs, including the reduction in anogenital distance, even at environmentally equivalent levels (Mably et al. 1992; Ohsako et al. 2001, 2010; Ko et al. 2004). These adverse effects depend on AHR and are due to the decreased sensitivity of the male genitalia to androgens (Ko et al. 2004; Ohsako et al. 2010). Furthermore, it has been reported that perinatal-stage TCDD exposure increased the basal level of SRD5A2 and decreased androgen receptor (AR) mRNA expression levels alongside upon CYP1 family transcriptional induction in the rat prostate (Theobald et al. 2000; Ohsako et al. 2001). Although the mechanism underlying this increase in SRD5A2 expression level is as yet unclear, epigenetic alteration in SRD5A2 may be a plausible explanation for the disorders of the male reproductive system (Horning et al. 2015; Skakkebaek et al. 2016; Mozhui and Pandey 2017). This suggests that the SRD5A2 mRNA expression level in the postnatal male genitalia may increase in the human population exposed to endocrine disrupting chemicals during the fetal or neonatal period.

In this study, to investigate the association between biomarker level as an index of chemical exposure and male infant reproductive disorders, we measured the mRNA expression levels of the CYP1A1 and CYP1B1 and the androgen-related genes (SRD5A2 and AR) in human preputial skin samples from patients with hypospadias and compared them with those from patients with phimosis. Additionally, we analyzed the SRD5A2 mRNA expression level and the promoter CpG methylation level to examine the interactions of epigenomic changes of SRD5A2 in the two male reproductive disorders.

## Materials and Methods

### Participants and Sample Preparation

Japanese patients with hypospadias (*n* = 23) and phimosis (*n* = 16) were studied. The ages of the patients are shown in Table S1. Preputial skin samples (approximately 1 mm^3^) of patients were collected at the time of surgery after obtaining their informed consent. Total RNA and DNA were purified from frozen skin samples using ISOGEN (Nippongene, Tokyo, Japan).

### Real-Time RT-PCR

The protocol of quantitative real-time RT-PCR analysis was as described previously (Ohsako et al. 2010). The primer sets used in this study are shown in Table S2. The target genes in the reverse-transcribed RNAs were amplified with SYBR Premix Ex Taq (Perfect Real Time) system (TaKaRa, Kusatus, Japan) using a LightCycler (Roche Manheim, Germany). The values were adjusted to cyclophilin-B (PPIB) level.

### Bisulfite Genomic Sequencing

The DNA was digested overnight with *Not* I restriction enzyme and then denatured with 0.3 M NaOH. Freshly prepared hydroquinone and sodium bisulfite were added to final concentrations of 0.5 mM and 3.1 M, respectively, and then the resulting mixture was incubated at 55 °C for 16 h. The CpG islands of target genes in the treated DNA were amplified by nested PCR using LA Taq DNA polymerase (TaKaRa). The primer sequences are shown in Table S3. PCR products were subcloned into pGEM-T Easy vector (Promega Corp., Madison, WI) to analyze the methylation pattern of each clone. DNA sequencing was performed by the dideoxynucleotide chain termination method using DYEnamic ET terminator cycle sequencing kit and Applied Biosystem 3730 DNA Analyzer (ThermoFisher Scientific Inc., San Diego, CA).

### Statistical Analysis

The expression levels of mRNAs were analyzed by the Mann–Whitney *U* test using StatView software (SAS Institute, USA). In correlation analysis, Pearson’s correlation coefficient was calculated, and no-correlation analysis was conducted using Microsoft Office Excel 2007 (Microsoft Co., USA). *P* < 0.05 was considered to indicate statistical significance.

## Results

### Differences in CYP1B1 and AR mRNA Expression Levels Between Hypospadias and Phimosis

**Fig. 1:**
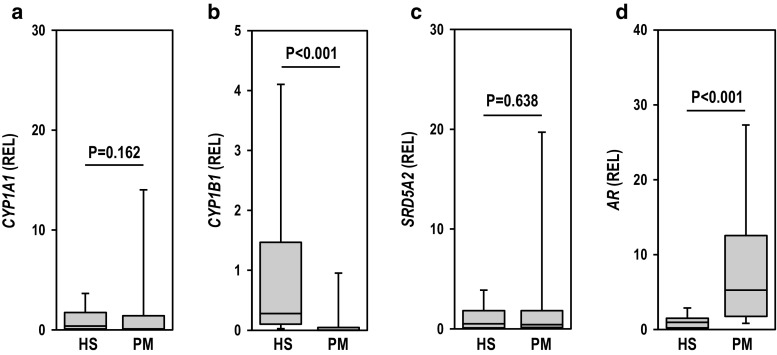
mRNA expression levels in patients with hypospadias (HS) and those with phimosis (PM). The mRNA expression levels of CYP1A1 (**a**), CYP1B1 (**b**), SRD5A2 (**c**), and AR (**d**) were measured by qRT-PCR analysis using a LightCycler. Values are shown as relative expression level (REL) to PPIB. Data were analyzed by the Mann–Whitney *U* test (HS, *n* = 23; PM, *n* = 16)

**Fig. 2:**
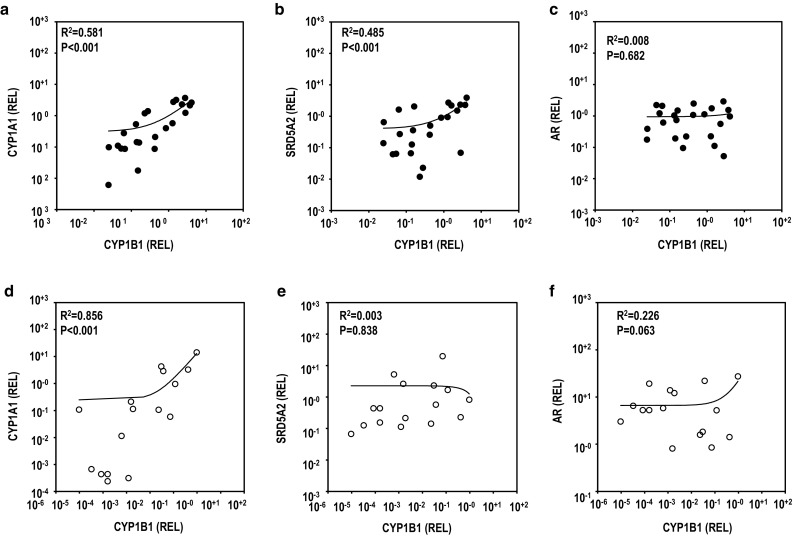
Correlation analysis of mRNA expression levels between CYP1B1 and other genes in hypospadias and phimosis patients. CYP1A1 in hypospadias (**a**) patients and phimosis (**d**) patients. SRD5A2 in hypospadias (**b**) and phimosis (**e**) patients. AR in hypospadias (**c**) and phimosis (**f**) patients. Pearson’s correlation analysis was performed (*p* < 0.05 was considered statically significant)

Although no significant difference in the expression level of CYP1A1 mRNA was observed between the hypospadias and phimosis groups (Fig. 1a), the expression level of CYP1B1 mRNA was significantly higher in the hypospadias group than in the phimosis group (Fig. 1b). The expression level of SRD5A2 mRNA in the hypospadias group was similar to that in the phimosis group (Fig. 1c); however, the AR mRNA expression level in the phimosis group was found to be higher than that in the hypospadias (Fig. 1d). These results indicate that the mRNA expression of the AR is probably down-regulated along with the up-regulation of the CYP1B1 gene in the skin of the external genitalia of patients with hypospadias. This is supported by the results of the correlation analysis described below (Fig. 2c).

### Correlation Between Expression Levels of CYP1 Family and SRD5A2 Gene

Because large deviations of expression levels were seen within each patient group, correlations of the mRNA expression levels were analyzed in more detail for each patient for all combinations of genes (Table S4). Of 13 human CYPs tested, the mRNA expression level of CYP1B1 has been reported to be much higher than those of CYP1A1 and CYP1A2 in an early study of healthy male volunteers (Yengi et al. 2003). Therefore, we focused on the correlations between the mRNA expression levels of the CYP1B1 gene and the other three genes in both patient groups (Fig. 2). The mRNA expression levels of CYP1A1 and CYP1B1 showed an extremely high correlation in both the hypospadias and phimosis groups (Fig. 2a, d). A significantly positive correlation was found between the mRNA expression level of SRD5A2 and that of CYP1B1 in the hypospadias group (Fig. 2b). However, no such correlation was found in the phimosis groups (Fig. 2e). A positive correlation also was seen between the mRNA expression level of SRD5A2 and that of CYP1A1 in the hypospadias group (Table S4). In both patient groups, the expression level of AR mRNA showed no significant correlation with that of CYP1B1 mRNA (Fig. 2c, f). These results suggest that in the hypospadias group, the SRD5A2 gene expression correlated with CYP1 family genes.

### CYP1 Family Expression Level Correlated with SRD5A2 Promoter Methylation Level

**Fig. 3:**
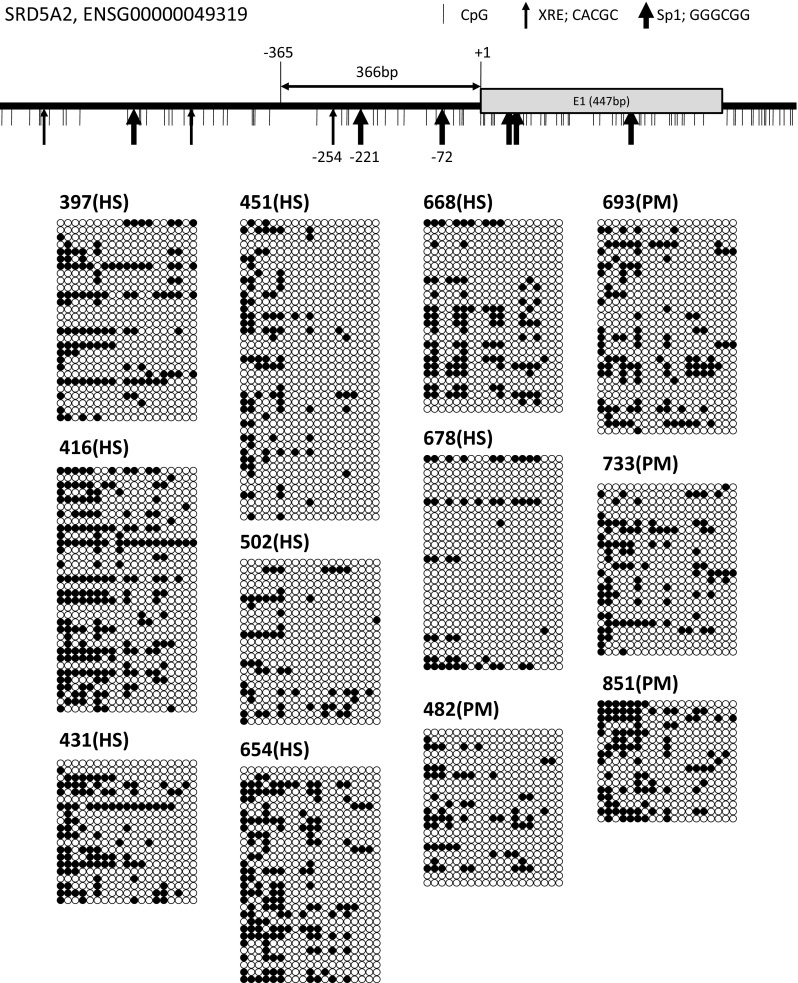
CpG island of SRD5A2 promoter region analyzed in this study and parts of bisulfite genomic sequencing results. Methylated CpG (closed circle), unmethylated CpG (open circle). Patient numbers are indicated in the upper left of bisulfite genomic sequencing results. *HS* hypospadias, *PM* phimosis

**Fig. 4:**
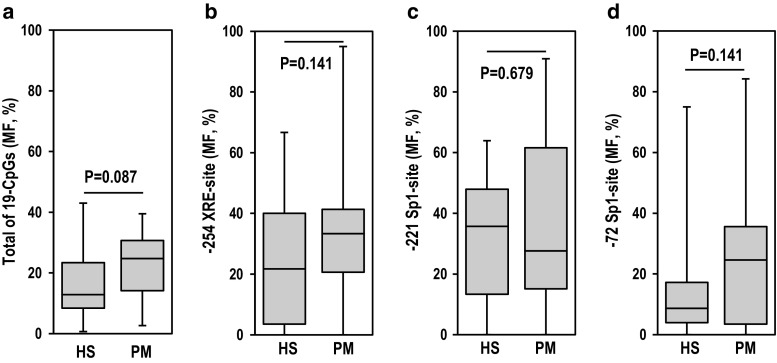
DNA methylation analysis by bisulfite genomic sequencing. Methylation frequency (MF, %) of a total of 19 CpGs − 532 to − 166 (**a**), − 254 XRE site (**b**), − 221 Sp1 site (**c**), and − 72 Sp1 site (**d**) of SRD5A2 gene promoter DNA were compared between hypospadias (HS) and phimosis (PM) patients. Data were analyzed by the Mann–Whitney *U* test (HS, *n* = 23; PM, *n* = 16)

**Fig. 5:**
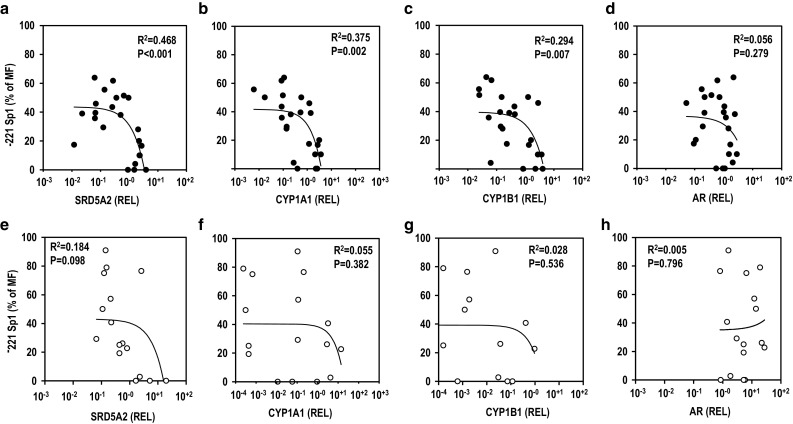
Analysis of correlation of methylation frequency (MF) of gene with mRNA levels in hypospadias and phimosis patients. Sp1 site at − 221 versus SRD5A2 in hypospadias (**a**) and phimosis (**e**) patients, Sp1 site at − 22 versus CYP1B1 in hypospadias (**b**) and phimosis (**f**) patients, Sp1 site at − 221 versus CYP1A1 in hypospadias (**c**) and phimosis (**g**) patients, and Sp1 site at − 221 versus AR in hypospadias (**d**) and phimosis (**h**) patients. Pearson’s correlation analysis was performed (*p* < 0.05 was considered statically significant)

Some bisulfite genomic sequencing results of CpG-islands in the SRD5A2 proximal promoter region are presented in Fig. 3. We examined the methylation level of the CpG islands of CYP1A1, CYP1B1, and AR genes; however, no methylation of these three genes was detected in all the samples examined (data not shown). No significant differences were found in the CpG methylation level of the promoter region of the SRD5A2 gene from − 365 to + 1 (total of 19 CpGs), the − 254 XRE site (CACGC), and the − 221 and − 72 Sp1 sites (GGGCGG) between the hypospadias and phimosis samples (Fig. 4a–d) . Correlation analyses between the SRD5A2 promoter DNA methylation level and the mRNA expression level were performed for all combinations of the four genes (Table S4). A significantly negative correlation was detected between the mRNA expression and methylation levels of SRD5A2 in the hypospadias group particularly at the − 221 Sp1 site, whereas no such negative correlation was detected in the phimosis group (Fig. 5a, e). Furthermore, a significantly negative correlation was detected between the mRNA expression levels of the CYP1 family genes and the methylation level of the − 221 Sp1 site of SRD5A2 in hypospadias (Fig. 5b, c). However, no such negative correlation was found in the phimosis patients (Fig. 5f, g). No significant correlation was detected between the AR mRNA expression level and the methylation level at the − 221 Sp1 site of the SRD5A2 gene in either group (Fig. 5d, h). These results showed that the mRNA expression levels of the CYP1 family genes significantly correlated with the mRNA expression level and DNA methylation level of SRD5A2 in patients with hypospadias.

## Discussion

In this study, using RNA samples clinically collected from preputial tissues, we first demonstrated that the expression levels of the xenobiotic exposure marker genes, CYP1A1 and CYP1B1 correlated only in hypospadias patients but not in phimosis patients. Additionally, using DNA prepared from identical tissues, we found a positive correlation between SRD5A2 gene DNA methylation levels and CYP1 family expression levels. These findings suggested the possible involvement of xenobiotics in the onset of hypospadias, including epigenetic modifications.

Administration of β-naphthoflavone, an AHR ligand, induces CYP1A1 enzymatic activity in the rat skin (Stauber et al. 1995). Epidermal cells of the human skin express CYP1A1 and CYP1B1 (Katiyar et al. 2000; Yengi et al. 2003). Therefore, the use of CYP1 family genes as biomarkers of the level of exposure to chemicals such as PAHs, OCs, and dioxin-like chemicals exposure seems reasonable even in the analysis of human skin samples. It is likely that the exposure level of patients with hypospadias is higher, as shown by the higher mRNA expression level of CYP1B1 in hypospadias patients than in phimosis patients, which suggests that exposure to environmental pollutants is associated with the onset of malformations of male reproductive system.

In utero and lactational exposure of pregnant rats and mice to dioxin causes abnormalities of male reproductive organs, such as the shortening of the anogenital distance or the delay of prostate development, in which an increase in prostatic mRNA SRD5A2 expression level is also observed (Theobald et al. 2000; Ohsako et al. 2001). Our preliminary rat studies revealed slight decreases of in the methylation levels of two CpGs in the minimal promoter of 5′upstream regions (− 75 to + 1) (Seenundun and Robaire 2005), suggesting that DNA methylation of SRD5A2 proximal promoter regions is implicated with transcription level (unpublished data). Because human SRD5A2 is highly synthesized in the genital skin of the fetal penis (Kim et al. 2002), SRD5A2 is considered to be another biomarker of chemical exposure in male genital tissues. In this study, we detected no significant differences in SRD5A2 mRNA expression level and SRD5A2 proximal promoter methylation level between the hypospadias and phimosis patients. However, we found significant correlations of CYP1 family mRNA expression levels with the SRD5A2 mRNA expression level and SRD5A2 promoter DNA methylation levels in the hypospadias patients, which suggests the involvement of chemical exposure in the onset of hypospadias. Additionally, the expression level of AR mRNA was found to be lower in the hypospadias group than in the phimosis group. This finding is similar to that of a previous study showing that the of prostatic AR mRNA expression level is decreased by in utero and lactational exposure to TCDD in rats (Ohsako et al. 2001).

Furthermore, our analysis showed that the methylation level of the SRD5A2 promoter region negatively correlated with not only the mRNA expression level of SRD5A2, but also those of the CYP1 family genes. Higher levels of CpG methylation at − 221/− 72, which is known as the Sp1 sites, co-occurred with the low level of SRD5A2 mRNA expression. In general, the Sp1 sites in promoter regions positively control the basal level of gene transcription (Kaczynski et al. 2003). In the case of rat SRD5A2, a study using luciferase reporter transfection demonstrated that the minimal promoter of the proximal promoter region from − 150 to + 1 containing Sp1 sites up-regulates SRD5A2 gene transcription (Seenundun and Robaire 2005). Therefore, the DNA methylation of this minimal promoter region seems to down-regulate SRD5A2 transcription.

Recently, genome wide methylation analysis using Illumina Infinium, with which 485,000 CpGs of promoter regions can be analyzed, has shown that the preputial tissue of patients with hypospadias did not show any significant difference in the methylation level in SRD5A2 from samples taken from healthy males, whereas SCARB1 and MYBPH hypermethylation was detected in the samples from the patients (Choudhry et al. 2012). However, the samples used in the study by Choudhry et al. (2012) were obtained from patients with a broad range of ages, which was different from our study. Further analysis using neonatal preputial tissues would be required to explore fully the pathogenesis of hypospadias caused by environmental factors.

The activity of SRD5A2 gradually decreases from 1 to 8 years of age in the frenulum skin of the human penis (Theintz et al. 1989). In this study, the SRD5A2 mRNA expression level in 1-to 2-year-old children was higher than that in older children (1–2 years, average = 6.14; 2–14 years, average = 0.29). Indeed, significant correlations were found between SRD5A2 methylation level and SRD5A2 mRNA expression level in the samples from 1- to 2-year-old children (data not shown). It is necessary to examine preputial tissues from normal healthy individuals to compare the mRNA expression levels of CYP1 family genes and the DNA methylation level of SRD5A2 with those in preputial tissues from individuals with hypospadias. Detailed analyses using physiological indices, such as the expression levels of other mRNAs and hormones, will provide more precise information about the onset of hypospadias caused by environmental factors.

## Conclusions

We found a significant positive association of mRNA expression level and a negative association of methylation level of the SRD5A2 gene with the mRNA expression levels of CYP1 family genes in the preputial tissue of patients with hypospadias. This seems to indicate the involvement of chemical exposure in the onset of hypospadias. The epigenomic alterations of the SRD5A2 gene may be associated with the activation of AHR induced by exogenous environmental chemicals.

## Electronic supplementary material

Below is the link to the electronic supplementary material.

Supplementary material 1 (PDF 147 kb)
