# Supplementary material for: Expression of Xenobiotic Biomarkers CYP1 Family in Preputial Tissue of Patients with Hypospadias and Phimosis and Its Association with DNA Methylation Level of SRD5A2 Minimal Promoter
Source: Arch Environ Contam Toxicol. 2017 Oct 27;74(2):240–7. doi: 10.1007/s00244-017-0466-x (PMC5807475; doi:10.1007/s00244-017-0466-x)
Supplement: Supplementary file 1 — Supplementary material 1 (PDF 147 kb) [file 244_2017_466_MOESM1_ESM.pdf]

Table S1 Patients participated in this study.

Phenotype	Patient no.	Age
Hypospadias	312	1 yr 8 m
	346	1 yr 10 m
	370	1 yr 6 m
	397	1 yr 7 m
	414	1 yr 11 m
	416	1 yr 5 m
	431	1 yr 6 m
	436	1 yr 10 m
	451	1 yr 7 m
	502	1 yr 6 m
	524	2 yr 7 m
	553	4 yr 1 m
	578	3 yr 1 m
	648	1 yr 11 m
	654	1 yr 7 m
	668	1 yr 6 m
	673	1 yr 7 m
	678	1 yr 11 m
	682	7 yr 3 m
	705	1 yr 2 m
	709	14 yr 0 m
	714	1 yr 1 m
	723	1 yr 9 m
Phimosis	116	1 yr 2 m
	228	1 yr 10 m
	239	1 yr 2 m
	308	1 yr 5 m
	395	1 yr 6 m
	399	1 yr 1 m
	463	1 yr 2 m
	480	1 yr 1 m
	631	8 yr 0 m
	693	3 yr 10 m
	697	1 yr 0 m
	733	2 yr 0 m
	738	1 yr 0 m
	749	1 yr 2 m
	851	1 yr 3 m
	896	1 yr 3 m

yr, years; m, months

Table S2 Primers used for quantitative RT-PCR analysis.

Gene	Primer sequences		Product size (bp)	GenBank accession no.
	Forward primer (5' ~ 3')	Reverse primer (5' ~ 3')		
CYP1A1	CCAGGCTCCAAGAGTCCACC	GCCTTTGGGGACCTGAGG	183	BC023019
CYP1B1	GGAGAACGTACCGGCCACTA	GCCAGGACATAGGGCAGGTT	198	NM_000104
AR	CGCTTCTACCAGCTCACCAA	AATGCTTCACTGGGTGTGGA	204	NM_000044.2
SRD5A2	CACCTGGGACGGTACTTCTG	GGACTCCATTTCCAGTGCAG	141	AF005204
PPIB	TCCGTCTTCTTCCTGCTGCT	AGCTAAGGCCACAAAATTATCCACT	180	NM_000942

Table S3 Primers used for bisulfite genomic sequencing.

Gene		Primer sequences		Product size (bp)
		Forward primer (5' ~ 3')	Reverse primer (5' ~ 3')	
SRD5A2	First	AGAGTTAGGATGGTTTAGGGTTTAA	ATTCRCAACAATACCCCTTTCTCAAAA	
	Nested	TTTAGGGTTTAAGGAAGGTTTTATGTT	AACAATACCCCTTTCTCAAAAATACAA	366
CYP1A1	First	ATTTTTGTGTTTTGTTAATTAAAGTATTAGTTATTT	AAAATCCCAAAACAACCCRAAAAAAAAATA	
	Nested	TTATTTYGGGAGTTAAGAGGGATTTTT	AAAAAAAAATACRAAAAACTCCATCCTAAAA	454
CYP1B1	First	AAAGAGTTTTTATTGAGGTGGTAAT	CACTCCCACTCCAAAATCAA	
	Nested	TTTTATTGAGGTGGTAATTTGTTTG	ACTCCCACTCCAAAATCAAAA	483
AR	First	GGAAGGTAAGGAGGTYGGTT	TCAATCCTACCAAACACTTTCCTTA	
	Nested	ATTYGTAAATTGTTGTATTTGTTTTT	TACCAAACACTTTCCTTACTTCCTC	324
	First	GGAAGTAAGGAAAGTGTTTGGTAG	AAAAACCTAACTACCTTTTCATCTTTT	
	Nested	AAGGAAAGTGTTTGGTAGGATTG	CCTTTTCATCTTTTAATCTCTAACTCC	423
	First	TTGTTGAGTATTTTTTTTTTAAGGGAG	TCCATACAACTAACCTTCTTCRAC	
	Nested	AGGGAGGTTATATTAAAGGGTTAGA	CAACTAACCTTCTTCRACTATAAAAA	401

Table S4 Correlation analyses among four mRNA levels, or between SRD5A2 promoter DNA methylation and the mRNA levels.

		Gene expression			*SRD5A2* promoter DNA methylation			
Phenotype		*CYP1B1*	*SRD5A2*	*AR*	Total 19-CpGs	-254 XRE-site	-221 Sp1-site	-72 Sp1-site
Hypospadias (n=23)	*CYP1A1*	r=0.762 p<0.001***	r=0.715 p<0.001***	r=0.133 p=0.545	r=-0.477 p=0.021*	r=-0.364 p=0.087	r=-0.612 p=0.002**	r=-0.306 p=0.156
	CYP1B1		r=0.697 p<0.001***	r=0.090 p=0.682	r=-0.523 p=0.011*	r=-0.547 p=0.007**	r=-0.542 p=0.007**	r=-0.296 p=0.171
	SRD5A2			r=0.333 p=0.121	r=-0.496 p=0.016*	r=-0.323 p=0.132	r=-0.684 P<0.001***	r=-0.446 p=0.033*
	AR				r=-0.007 p=0.974	r=0.042 p=0.849	r=-0.236 p=0.279	r=0.068 p=0.759
Phimosis (n=16)	*CYP1A1*	r=0.925 p<0.001***	r=-0.112 p=0.680	r=0.568 p=0.022*	r=0.187 p=0.488	r=-0.029 p=0.914	r=-0.235 p=0.382	r=-0.205 p=0.447
	CYP1B1		r=-0.055 p=0.838	r=0.475 p=0.063	r=0.201 p=0.456	r=0.045 p=0.868	r=-0.167 p=0.536	r=-0.226 p=0.400
	SRD5A2			r=-0.293 p=0.271	r=-0.548 p=0.028*	r=-0.233 p=0.385	r=-0.429 p=0.098	r=-0.386 p=0.140
	AR				r=0.457 p=0.075	r=0.045 p=0.869	r=0.070 p=0.798	r=0.315 p=0.234

The mRNA levels of four genes (*CYP1A1*, *CYP1B1*, *SRD5A2*, and *AR*) in the skin specimens from the patients with hypospadias and phimosis were measured by qRT-PCR using LightCycler. DNA methylation frequency of *SRD5A2* gene were analyzed by bisulfite genomic sequencing. The values of relative expression levels (REL) to PPIB and methylation frequency (%) were used in the above analyses. Pearson's correlation analysis was performed. *, p<0.05; **, p<0.01; ***, p<0.001.
